# A Novel Peptide ELISA for Universal Detection of Antibodies to Human H5N1 Influenza Viruses

**DOI:** 10.1371/journal.pone.0020737

**Published:** 2011-06-10

**Authors:** Sumathy Velumani, Hui-Ting Ho, Fang He, Syed Musthaq, Mookkan Prabakaran, Jimmy Kwang

**Affiliations:** 1 Animal Health Biotechnology, Temasek Life Sciences Laboratory, National University of Singapore, Singapore, Singapore; 2 Department of Microbiology, Faculty of Medicine, National University of Singapore, Singapore, Singapore; Centers for Disease Control and Prevention, United States of America

## Abstract

**Background:**

Active serologic surveillance of H5N1 highly pathogenic avian influenza (HPAI) virus in humans and poultry is critical to control this disease. However, the need for a robust, sensitive and specific serologic test for the rapid detection of antibodies to H5N1 viruses has not been met.

**Methodology/Principal Findings:**

Previously, we reported a universal epitope (CNTKCQTP) in H5 hemagglutinin (HA) that is 100% conserved in H5N1 human isolates and 96.9% in avian isolates. Here, we describe a peptide ELISA to detect antibodies to H5N1 virus by using synthetic peptide that comprises the amino acid sequence of this highly conserved and antigenic epitope as the capture antigen. The sensitivity and specificity of the peptide ELISA were evaluated using experimental chicken antisera to H5N1 viruses from divergent clades and other subtype influenza viruses, as well as human serum samples from patients infected with H5N1 or seasonal influenza viruses. The peptide ELISA results were compared with hemagglutinin inhibition (HI), and immunofluorescence assay and immunodot blot that utilize recombinant HA1 as the capture antigen. The peptide ELISA detected antibodies to H5N1 in immunized animals or convalescent human sera whereas some degree of cross-reactivity was observed in HI, immunofluorescence assay and immunodot blot. Antibodies to other influenza subtypes tested negative in the peptide-ELISA.

**Conclusion/Significance:**

The peptide-ELISA based on the highly conserved and antigenic H5 epitope (CNTKCQTP) provides sensitive and highly specific detection of antibodies to H5N1 influenza viruses. This study highlighted the use of synthetic peptide as a capture antigen in rapid detection of antibodies to H5N1 in human and animal sera that is robust, simple and cost effective and is particularly beneficial for developing countries and rural areas.

## Introduction

H5N1 highly pathogenic avian influenza (HPAI) virus remains an important global concern as it has caused devastating outbreaks in poultry worldwide, and over 500 human cases with a substantially high fatality rate of 60% [1]. If H5N1 could exhibit sustained transmissions among humans, an unusually severe pandemic threat would be inevitable.

An effective surveillance system for H5N1 infection in animals and humans is of vital importance in the control of this disease. Currently available serologic tests include hemagglutination inhibition (HI), microneutralization assay, enzyme-linked immunosorbent assay (ELISA) and agar gel precipitation test. The HI test is considered the “gold standard” for subtyping. However, low sensitivity and subtype cross-reactivity significantly limit the value of this assay [2], [3]. Although the microneutralization assay overcomes these limitations and is currently the recommended test for H5N1 infection, this test is labour-intensive and requires biocontainment facilities [4]. A commercial ELISA allows rapid serologic surveillance of H5N1 infection has been reported. However, false positive results were observed in cross-reacting antibodies of seasonal influenza viruses [5].

Peptide based enzyme immunoassay (ELISA) has been widely used for the sero-diagnosis of bacterial and viral infections [6], [7]. It provides the advantage of enhanced specificity and can be easily implemented into a simple, rapid, sensitive and relatively cheap diagnostic kit [8], [10], [11]. Previously, we reported an immunogenic epitope comprising the sequence “CNTKCQTP” that is highly conserved in the H5 hemagglutinin (HA) [9]. Antibodies to this epitope were readily detected in H5N1 immunized animals or convalescent human sera in the format of epitope-blocking ELISA. However, this epitope-blocking ELISA requires the presence of a H5 antigen and relies on the specific monoclonal antibody that binds to the epitope. As a result, it is relatively expensive and impractical for development into a rapid diagnostic kit that is suitable for field use.

In the present study, we developed a novel peptide ELISA for H5N1 antibody detection by incorporating the peptide comprising the amino acid sequence “CNTKCQTPMGAINSS”. The specificity of the peptide ELISA was assessed by experimental chicken antisera against multiple clades of H5N1 viruses, including clades 0, 1, 2.1, 2.1.1, 2.1.2, 2.1.3, 2.2, 2.3, 4, 7 and 8, as well as other subtype influenza A viruses and convalescent human sera. Furthermore, we demonstrate the superior specificity of the peptide ELISA in comparison with hemagglutinin inhibition (HI), dot blot and immunofluorescence assay (IFA) that utilize recombinant HA1 proteins as diagnostic antigen. In addition, this method is a simple, highly sensitive and specific for a particular subtype H5N1. In future this method could be a good serodiagnosis method for effective replacement of serum neutralization which needs heightened containment and live virus.

## Materials and Methods

### Viruses

Influenza A viruses isolated in Indonesia were obtained from Ministry of Health (MOH) (Table 1). Influenza A viruses from other subtypes were obtained from Agri-food and Veterinary Authority (AVA) of Singapore (Table 2). Reverse genetics system has allowed the generation of reassortant viruses containing HA and NA from different clades of H5N1 and other subtype viruses in A/Puerto Rico/8/1934 (PR8) genetic background [12] (Table 1, 2). High and Low pathogenic viruses were propagated in 11-day old embryonated chicken eggs and virus titers were determined by hemagglutinin assay [13]. Viruses were clarified by centrifugation at 13000×g for 15 min and inactivated using binary ethylenimine (BEI) to a final concentration of 0.01 M for 24 h at 37°C [14] and stored at −80°C. All experiments with live viruses were performed in a biosafety level 3 containment laboratory in compliance with CDC/NIH and WHO recommendations [15] and also approved by the Agri-food and Veterinary agency and Ministry of Health of Singapore.

**Table 1:** H5N1 subtype influenza A viruses used in this study.

Strain	Subtype	Clade	Host
A/Hongkong/156/97*	H5N1	0	human
A/Honglong/213/03*	H5N1	1	human
A/Indonesia/CDC1031/07*	H5N1	2.1	human
A/Egypt/0636-NAMRU3/07*	H5N1	2.2	human
A/Nigeria/6e/07*	H5N1	2.2	human
A/Anhui/1/05*	H5N1	2.3	human
A/Vietnam/HN/31242/07*	H5N1	2.3	human
A/Indonesia/CDC7/06	H5N1	2.1.1	human
A/Indonesia/CDC523/06	H5N1	2.1.3	human
A/Indonesia/CDC594/06	H5N1	2.1.2	human
A/Indonesia/CDC669/06	H5N1	2.1.3	human
A/goose/Guiyang/337/06*	H5N1	4	avian
A/chicken/Shanxi/2/06*	H5N1	7	avian
A/chicken/Henan/12/04*	H5N1	8	avian

*Reassortant viruses generated from reverse genetics.

**Table 2:** Influenza A viruses H1–H13 and H16 subtypes used in this study for specificity evaluation.

Strain	Subtype	Host
A/Brisbane/59/2007*	H1N1	human
A/Singapore/TLL52/2009*	H1N1	human
A/duck/Nanchang/4-184/2000*	H2N9	avian
A/Chicken/Singapore/02	H3N2	avian
A/Chicken/Singapore/92	H4N1	avian
A/Hongkong/213/03*	H5N1	human
A/Shorebird/DE/12/04*	H6N8	avian
A/Chicken/Singapore/94	H7N1	avian
A/duck/Yangzhou/02/05*	H8N4	avian
A/chicken/Singapore/Sin/98	H9N2	avian
A/mandarin duck/Singapore/Sin/98	H10N5	avian
A/pintail/Alberta/84/2000*	H11N9	avian
A/pintail/Alberta/49/03*	H12N5	avian
A/gull/Maryland/704/1977*	H13N6	avian
A/herring gull/Delaware/712/1988*	H16N3	avian

*Reassortant viruses generated from reverse genetics.

### Reverse Genetics

The HA and NA genes of 10 H5N1 viruses from clade 0, 1, 2.1, 2.1.1, 2.1.2, 2.1.3, 2.2, 2.3, 4, 7 and 8 as well as eight non-H5 subtypes viruses (H1N1, H2N9, H6N8, H8N4, H11N9, H12N5, H13N6 and H16N3) (Table 1,2) were synthesized by GenScript based on the sequences from the NCBI influenza database. The synthesized HA and NA genes were cloned into a dual-promoter plasmid for influenza A reverse genetics [12]. Reassortant viruses were rescued by transfecting plasmids containing HA and NA together with the remaining six gene plasmids derived from A/Puerto Rico/8/34 (H1N1) into co-culture of 293T and MDCK cells by using Lipofectamine 2000 (Invitrogen Corp). At 72 h post transfection, culture medium from the transfected cells was inoculated into 11-day-old embryonated chicken eggs or MDCK cells. The HA and NA genes of reasssortant viruses were sequenced to confirm the presence of introduced HA and NA genes.

### Experimental serum samples

Experiments involved animals were undertaken in a biosafety level 3 facilities at Temasek Lifesciences Laboratory, Singapore. Animals were handled according to Institutional Animal Care and Use Committee (IACUC) regulations. Inactivated H5N1 and other subtype viruses (Table 1 and 2) were emulsified in ISA-70 (SEPPIC, France) adjuvant and injected intramuscularly to the groups of three weeks old white leghorn chickens (n = 4). The booster was given twice at two-week intervals. Sera were prepared from the blood collected 10 days after 1st injection and 2^nd^ injection. Antibody responses to the homologous strains were evaluated by HI as described below.

### Human serum samples

Three serum panels from human (n = 50) were used. First panel comprised human sera from 15 individuals with confirmed H5N1 infection collected from the National institute of Hygiene and Epidemiology, Vietnam and MOH of Indonesia. Three out of 15 samples were collected 2 days after the onset of infection (collection date of other samples was unknown). Second panel comprises 15 human sera from patients recovered from seasonal influenza A virus infections from Singapore General Hospital (SGH) (no details on the collection date were provided). The third panel consisted of 20 serum samples from healthy volunteers with no influenza vaccination or any illness related to influenza, obtained from Ministry of Health (MOH) of Indonesia (n = 20).

### Immunofluorescence assay

A recombinant baculovirus harboring the HA1-encoding region (amino acids 1–325) from the A/goose/guangdong/2006 HA gene was generated as described previously [16], [17] Sf-9 cells were seeded in 96-well plates and infected with H5-HA1 recombinant baculovirus. At 36 h post-infection, the cells were fixed with 4% paraformaldehyde for 30 min at room temperature. All the washes were performed with phosphate buffered saline (PBS). Fixed cells were incubated with experimental chicken sera in 1∶250 dilution at 37°C for 1 h, washed and incubated with a 1∶40 dilution of fluorescein isothiocyanate (FITC)- conjugated goat anti-chicken IgG (Dako, Denmark). Antibody binding was visualized using Olympus IX 71 microscope (OLYMPUS, Japan) with appropriate barrier and excitation filters [18].

### Production of recombinant HA1 protein (rHA1)

Viral RNA was isolated with TRIzol (invitrogen,USA) from allantoic fluid containing H1–H16 subtype viruses (except H14 and H15 due to their rarity). The HA1 genes were amplified and cloned into pET -28a vector (Novagen, Germany) followed by transformation into *Escherichia coli* BL-21 competent cells for HA1 expression. Hexa-histidine tagged fusion HA expression was induced by the addition of 1 mmol/L IPTG for 4 h and purified on a Nickel-NTA column (Qiagen, Germany) [19].

### Hemagglutinination inhibition assay

Hemagglutination inhibition (HI) assay was performed as described previously [20]. Briefly, receptor destroying enzyme (RDE) treated sera were serially diluted (2 fold) in V bottom 96-well plates. Approximately 8 HA units of viral antigens were incubated with the sera for 30 min at room temperature, followed by the addition of 1% chicken red blood cells. The HI titre was the highest serum dilution in which agglutination was not observed.

### Immunodot blot

Purified proteins H1–H13, and H16 rHA1 proteins (1 µg) were blotted onto the 0.45 mm nitrocellulose membrane (Bio-rad) using a 96-well hybridot manifold (BIORAD). The membrane was then blocked with 5% nonfat milk in PBS containing 0.1% tween 20 (PBST) at room temperature for 30 min. All the rinsing was done with PBST for 3 times. The membrane was further incubated with diluted chicken sera (1∶250) in PBST at room temperature for 1 hr. Following rinsing, the membrane was incubated with horseradish peroxidase- conjugated goat anti-chicken IgG (Kirkegaard & Perry Laboratories, Gaithersberg, Md.) for 1 hr at room temperature. Bound antibodies were detected by the addition of DAB and H_2_O_2_ as the substrate, as described previously [21].

### Microneutralization assay

This assay describes the serum neutralizing antibody titers [3]. Briefly, MDCK cells were seeded at 1×10^6^ cells/well in 96-well culture plates and cultured at 37°C to form a monolayer. Serial two fold dilutions of RDE treated sera were mixed separately with 100 TCID_50_ of H5N1 strain incubated at room temperature for 1 h, and added to the monolayer of MDCK cells in triplicate wells. The neutralizing titers reported are the highest serum dilution in which no cytopathic effect was observed.

### Epitope-Blocking ELISA

Epitope-blocking ELISA (EB-ELISA) was performed as described before [9]. Briefly, U-bottomed 96-well ELISA plates were coated with purified recombinant HA of A/Indonesia/CDC/669/06 (500 ng/well) or inactivated concentrated H5N1 virus (10 µg/well) and incubated overnight at 4°C in coating buffer (0.1 mol/L carbonate/bicarbonate, pH 9.6). Antigen-coated plates were washed with PBS containing 0.05% Tween 20, pH 7.5 (PBST) and non-specific sites were blocked with 100 µL blocking buffer (PBS containing 5% skim milk) for 40 min at 37°C. Serially diluted (two-fold) test serum samples (100 µL) was added to each well and incubated for 45 min at 37°C. The wells were rinsed four times with PBST and incubated with 150 ng of HRP conjugated anti-H5 monoclonal antibody (mAb) 5F8 in 100 µL PBST for 1 h at 37°C. Following rinsing with PBST, signal was developed with 100 µL of 3, 3, 5, 5-tetramethyl benzidine (TMB, Sigma, USA) and the reaction was stopped by adding 0.1N Sulfuric acid. The optical density (OD) was determined at 450 nm using a multiwell plate reader. Serum samples show >30% inhibition is considered positive.

### H5 peptide ELISA

Peptide of amino acid sequence “CNTQCNTPMGAINSS” used in the ELISA was synthesized by PEPNOME Pte Ltd, China. Flat bottom 96-well amino immobilizer plates (NUNC, USA) were coated with 10 µg/well of peptide in coating buffer (10 mM NaHCO_3_ buffer, pH 9.6) and incubated at 4°C overnight. Plates were washed 3 times with PBST after each incubation. Peptide-coated plates were blocked by incubation with 200 µL blocking buffer (1% BSA in PBST) at 37°C for 1 h, and incubated either with 100 µL of chicken antisera or with anti-human serum in blocking buffer at 37°C for 45 min. Antibody binding was detected by incubation with 100 µL of species specific HRP conjugates (Kirkegaard & Perry Laboratories, Gaithersberg, Md.) diluted 1∶2000 in blocking buffer at 37°C for 45 min. The chromogen development was mediated by the addition of 100 µL freshly prepared substrate solution (3, 3′, 5, 5′-tetramethylbenzidine, Sigma). The reaction was stopped by addition of 50 µL of 4 M sulfuric acid and the OD was measured at 450 nm.

## Results

### Antisera to H1-H13 and H16 subtypes and H5N1 clades

A panel of antisera to influenza A viruses of subtype H1 to H13, and H16 was raised to determine the specificity of the peptide ELISA to H5 antibodies (Table 2). Reverse genetics was used to rescue viruses from H5N1 clades or HA subtypes that were not available in our laboratory as reassortants harboring internal genes (PB1, PB2, PA, NP, M and NS) from A/Puerto Rico/8/34. Ten H5N1 reassortant viruses habouring HA and NA of H5N1 viruses from clades 0, 1, 2.1, 2.1.1, 2.1.2, 2.1.3, 2.2, 2.3, 4, 7 and 8 (Table 1) and eight additional subtypes H1N1,H2N9, H6N8, H8N4, H11N9, H12N5, H13N6 and H16N3 (Table 2) were generated. These viruses were used to raise antisera in chickens to evaluate the peptide ELISA. The antisera were standardized to HI titre of 128 using the homologous viruses (Table 3).

**Table 3:** Cross-HI test with antiserum obtained from immunized chickens.

Antigen	Antiserum Titer^a^													
	H1	H2	H3	H4	H5	H6	H7	H8	H9	H10	H11	H12	H13	H16
H1N1^b^	128	-	8	-	-	8	-	-	-	-	-	4	-	-
H2N9	-	128	-	-	16	-	-	8	-	-	-	4	-	-
H3N2	-	-	128	4	-	-	-	-	2	-	-	-	-	-
H4N1	-	-	4	128	-	-	-	-	-	-	-	-	-	-
H5N1	-	8	-	-	128	-	-	-	2	-	-	-	-	2
H6N8	-	4	-	-	-	128	-	-	-	-	-	-	-	-
H7N1	-	-	-	-	-	-	128	-	-	-	-	-	-	-
H8N4	-	-	-	-	-	4	-	128	-	-	-	-	-	-
H9N2	-	-	2	-	-	-	-	-	128	-	-	-	-	-
H10N5	-	-	-	-	-	-	-	-	-	128	-	-	-	-
H11N9	-	-	-	-	-	-	-	-	-	-	128	-	-	-
H12N5	8	-	-	-	-	-	-	-	-	-	-	128	-	-
H13N6	-	-	-	-	-	-	-	-	-	-	-	-	128	-
H16N3	-	-	-	-	4	-	-	-	-	-	-	-	-	128

a The HI titer of the sera were adjusted to 128 with the homologous vírus (Table 2).

b A/Singapore/TLL52/2009*.

“-” NIL.

### Cross HI test

To determine the specificity of the HI test in identification of subtype-specific antibodies, the HI test was performed with H1–H13 and H16 antigens (Table 3). Generally, cross-reaction of H5 antiserum with heterologous antigen was minimal and negligible (≤1∶4). However, H5 antiserum and H2 antigen exhibited high cross-reactivity (1∶16). The cross-reactivity between these two subtypes is because of the relatively high HA sequence similarity [22].

### Immunofluorescence assay

**Figure 1:**
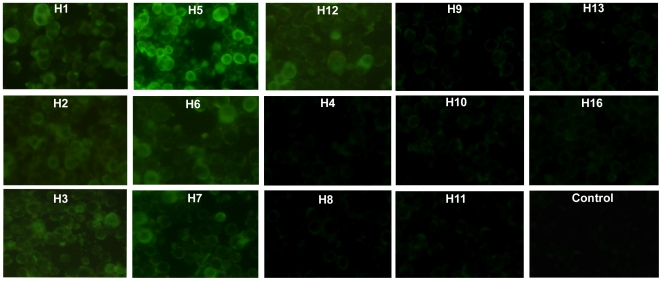
Immunofluorescence assay using Sf-9 cells infected with baculovirus harbouring HA1 of A/Goose/Guangdong/H5N1. After 36 hrs of infection, the cells were fixed and incubated with experimental chicken H1–H13 and H16 antisera and binding was detected with FITC-conjugated goat anti-chicken IgG. Strong fluorescence signal was observed with all experimental raised anti-H5 sera (only representative image is shown). Anti- H1, H2, H3, H6, H7 and H12 showed weak fluorescence signal, indicating some degree of cross reactivity. Antisera of other subtypes did not bind to the H5 HA1antigen. Control chicken serum (c) was also included as a negative control.

To determine the specificity of using H5 HA1 antigen in identification of subtype-specific antibodies, indirect immunofluorescence assay with sf-9 cells infected with recombinant baculovirus expressing H5 HA1 was conducted against H1–H13 and H16 antisera. Antisera to different H5N1 viruses yielded strong cytoplasmic immunoflurosence (Fig. 1 shows the representative images, other data not shown). Cross-reactivity was observed with antibodies of subtype H1, H2, H3, H6, H7 and H12 (Fig. 1). The positive signal from these non-H5 subtypes could be explained by the sequence homology with H5 and also >40% amino acid identity among influenza A subtypes [23], [24].

### Immunodot blot

**Figure 2:**
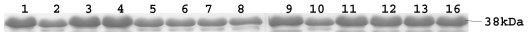
Separation of purified recombinant HA1 proteins of H1–H13 and H16 [**14**] subtypes (38 kDa) in SDS-PAGE.

**Figure 3:**
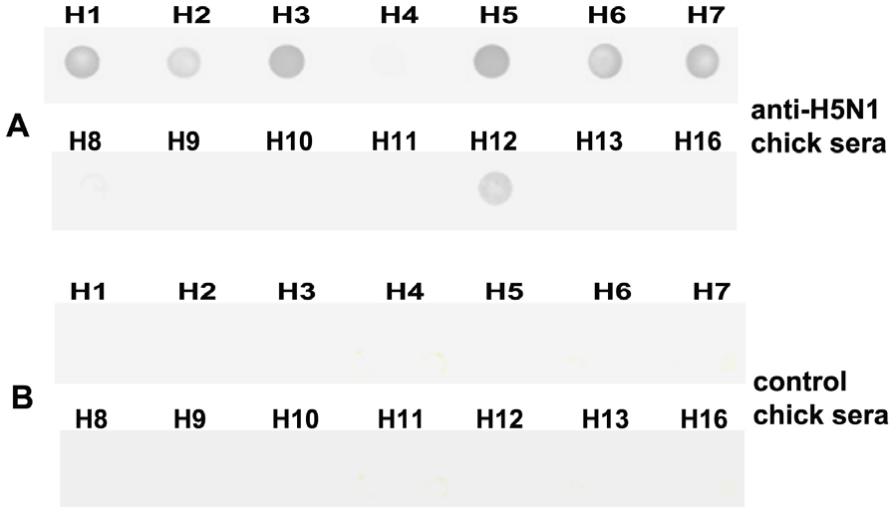
Recognition of H1–H13 and H16 rHA1 proteins by anti-H5N1 (A/Indonesia/CDC669/06-a representative for H5N1) chicken sera in immunodot blot. The membrane was blotted with column purified HA1 proteins of different subtypes, followed by incubation with anti-H5N1 chicken sera (A) or control chicken sera (B), and binding was evaluated with HRP-conjugated anti-chicken IgG.

With the advantage of eliminating HA2 that presents in HI test, we conducted immunodot blot assay using purified HA1 proteins of subtype H1–H13 and H16 to determine the specificity of HA1 in the identification of subtype-specific antibodies (Fig. 2). The H5 antisera reacted strongly to H5 HA1 but cross reactivity was observed with HA1 of H1, H2, H3, H6, H7 and H12 subtype (Fig. 3).

### Development of H5 peptide ELISA

**Figure 4:**
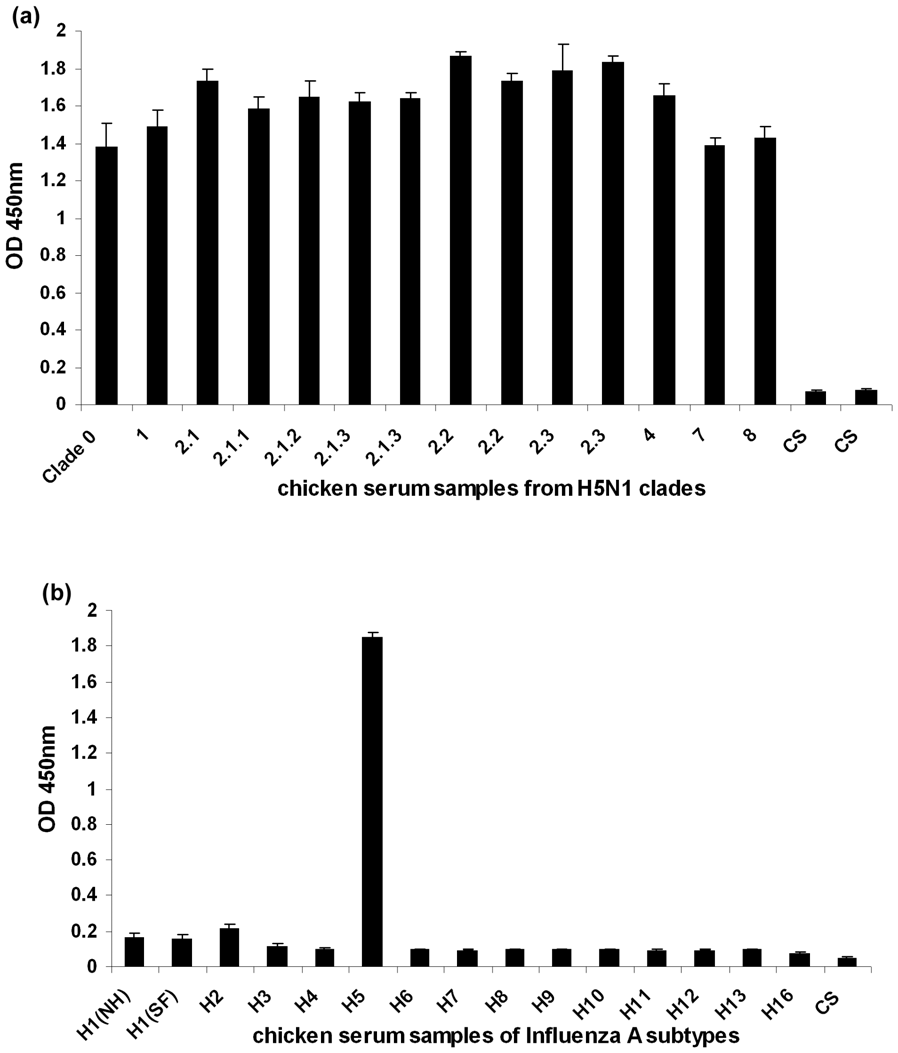
H5 peptide ELISA was performed with sera from chickens immunized with (a) H5N1 virus strains from different clades, covering clade 1, 2.1, 2.1.1, 2.1.2, 2.1.3, 2.2, 2.3, 0, 7, 4 and 8 (b) influenza A viruses H1–H13 [H1(NH)- new human & (SF)- seasonal flu)] and H16 subtypes. Sera were adjusted to HI titer of 16 to homologous viruses and diluted 1∶100 prior to use for analysis. The results were expressed as the mean of absorbance value (n = 4/group, whiskers above bars represent the standard deviation of the mean). CS: pre-immune chicken serum.

The peptide of amino acids sequence “CNTQCNTPMGAINSS” incorporated in the H5 peptide ELISA comprises the highly conserved and antigenic epitope (CNTQCNTP) of H5 HA as published in our previous study [9]. The specificity and sensitivity of the H5 peptide ELISA was examined using antisera from experimentally immunized chickens. Chicken sera collected 10 days after the 2nd immunization were adjusted to HI titer of 16 with the homologous virus to normalize antibody concentration and further diluted 1∶100 prior to use in H5 peptide ELISA. Figure 4a shows the absorbance values of H5N1 virus strains from different clades, covering clade 0, 1, 2.1, 2.1.1, 2.1.2, 2.1.3, 2.2, 2.3, 4, 7 and 8. The signal was considered to be positive when the absorbance was 3 times higher than that of the control serum. Absorbance values of all H5N1 serum samples were significantly higher than control serum and at least 5 times higher than that of the non-H5 subtypes viruses, with an average absorbance of 1.6 (Fig. 4a). No cross-reactivity was observed for any of the non-H5 subtype sera tested, and the average absorbance readings was 0.089 (Fig. 4b). These results indicate that the H5 peptide ELISA could positively identify serum samples containing antibodies to H5 and tested negative for sera containing antibodies to other HA subtypes.

### Human serum samples

To determine whether the peptide “CNTQCNTPMGAINSS” in the H5 peptide ELISA could recognize antibodies elicited by natural H5N1 infection in humans, convalescent serum samples from patients with laboratory-confirmed H5N1 infection were evaluated. A total of twelve out of fifteen serum samples obtained from patients from Vietnam and Indonesia tested positive in H5 peptide ELISA, with absorbance values of at least 5 times higher than the average absorbance value of normal human sera (0.0739) (Table 4). Even though some of these human serum samples showed a low HI titer of 4–16 only (Table 4), these serum samples were tested positive in EB-ELISA with mAb 5F8, indicating that the sensitivity of H5 peptide ELISA is comparable to that of EB-ELISA (Table 4). In addition, serum neutralization titer showed a comparative result to EB-ELISA and Peptide-ELISA (Table 4). Three serum samples collected 2 days after the onset of infection, when antibodies had not been produced were tested negative in serum neutralization, EB-ELISA and peptide-ELISA (Table 4). However, these samples showed an unspecific HI titre of 8–16. Serum samples from patients recovered from seasonal influenza A virus infection yielded negative results in H5 peptide ELISA, with a maximum absorbance of 0.091 (Table 4). However, some of these sera showed a HI titer of 8 against H5N1 virus, indicating some degree of cross-reactivity (Table 4). These data further demonstrate the reliability of H5 peptide ELISA in the detection of H5 antibodies in serum samples and minimal cross-reactivity due to antibodies elicited by seasonal influenza viruses.

**Table 4:** Analysis of human serum samples in H5 epitope-ELISA.

Human samples	HI assay	SN	EB-ELISA≠	Pep-ELISA*Mean ± SD	Pep-ELISA
H5N1 infected samples					
Vietnam					
31209	128	>1280	P	0.899±0.007	P
31203	128	>1280	P	0.858±0.006	P
30402	64	>640	P	0.712±0.007	P
31244	4–8	80	P	0.472±0.0071	P
30583	16	160	P	0.513±0.0071	P
30768	16–32	320	P	0.626±0.006	P
30777	16	320	P	0.530±0.007	P
31D-11	16	320	P	0.653±0.007	P
∧ELI 138	8	20	N	0.121±0.006	N
∧30406	16	40	N	0.132±0.007	N
∧CM1541	8	20	N	0.119±0.006	N
Indonesia					
292	32	640	P	0.658±0.0051	P
554	32	>320	P	0.598±0.006	P
645	16	160	P	0.555±0.006	P
828	16	160	P	0.549±0.007	P
Seasonal influenza infection samples					
Singapore					
9115	8	20	N	0.085±0.006	N
9129	<8	20	N	0.0723±0.007	N
9873	<8	20	N	0.0810±0.008	N
9106	8	<40	N	0.089±0.007	N
9708	<8	20	N	0.0735±0.006	N
9824	<8	20	N	0.0712±0.006	N
9872	8	<40	N	0.0867±0.006	N
9624	<8	20	N	0.0698±0.007	N
9792	<8	20	N	0.0714±0.007	N
9140	<8	20	N	0.0724±0.007	N
9151	8	20	N	0.0731±0.007	N
9159	<8	20	N	0.0743±0.005	N
9216	<8	20	N	0.0722±0.007	N
9286	8	<40	N	0.0854±0.007	N
9398	8	<40	N	0.0910±0.006	N
Normal healthy controls					
N = 20	0.0	0.0	N	0.0739±0.04	N

≠ Inhibition above the cut-off value of ≥30% blocking was considered positive (P).

*mean ± standard deviation of duplicate values of 2 independent experiments.

∧ Serum collected 2 days after the onset of infection.

P: positive, N: negative.

## Discussion

Successful control of H5N1 HPAI viruses requires active serological surveillance in animals and humans. Despite the limitations of conventional methods such as HI and microneutralization assay for serological surveillance, the main drawback of these methods is their impracticality for field investigation. We reported an EB-ELISA for detection of H5 antibodies based on a mAb that recognizes a highly conserved and antigenic epitope “CNTKCQTP” in influenza H5 HA [9]. However, this format is costly and less ideal to be developed into a rapid diagnostic kit.

Peptide ELISA has been used for sensitive and specific detection of biomarker antibodies in the diagnosis of many diseases [6], [25], and antibodies to other viral pathogens [7], [26]. To develop a robust, simple and cost-effective diagnostic tool for rapid H5 antibody detection that is suitable for field use, we incorporated the H5 HA specific epitope (CNTKCQTP) into peptide ELISA format. Thorough characteristic of this epitope in all influenza A viruses in our previous study showed that the conservation is 100% in all human H5N1 viruses and near complete absence in other subtypes [9]. In addition, the data from both our studies showed that this epitope is highly antigenic as antibodies to this epitope were readily detected in sera from chickens immunized with divergent clades of H5N1 viruses. Furthermore, in agreement with the characteristics of the epitope, the results showed that the H5 peptide ELISA based on the sequence of this epitope was 100% specific in the detection of H5 antibodies.

Some degree of cross-reactivity was observed in HI assay, and the immunodot blot and immunofluorescence assay that utilized HA1 proteins as the antigens for the detection of H5 antibodies. These observations are expected as number of studies have reported invariable conserved region between different subtypes of HA protein [3], [22], [27], [28]. Therefore, it further indicates the advantage and essential of the peptide approach to eliminate homologous epitopes between subtypes in order to achieve near absolute specificity.

The performance characteristic of the H5 peptide-ELISA was evaluated with human serum specimens with confirmed H5N1 infection history. In agreement with results obtained from experimental immunized chicken antisera, the H5 peptide-ELISA detected H5N1 antibodies in convalescent human sera. In addition, no false-positive test was observed with convalescent sera from seasonal influenza virus infection. We reported the high sensitivity of EB-ELISA [9], and the H5 peptide-ELISA in this study demonstrated comparable sensitivity with that of EB-ELISA. With the limited number of human serum samples from confirmed H5N1 individuals in this study, the H5 peptide-ELISA exhibited 100% sensitivity (n = 12) and 100% specificity (n = 35).

In summary, the H5 peptide-ELISA developed in this study provides sensitive and highly specific detection of H5 antibodies. Although the serum neutralization is considered as the gold standard for serodiagnosis, it has some drawbacks of time consuming and the need of heightened containment and use of live virus. The data suggests that this H5 peptide-ELISA based on the highly conserved and antigenic H5 HA epitope “CNTKCQTPMGAINSS” could be formatted as a rapid field test based on dipstick or lateral flow technologies, which greatly facilitate field investigation. As suggested by the global strategy for prevention and control of H5N1 HPAI, every country should have a surveillance system for detecting the virus in poultry [29]. Effective control of HPAI in poultry is critical as it limits the exposure of humans to the virus, thus diminishing the opportunities of the development of novel virus that acquires the ability to transmit from human to human [29]. Rapid diagnostic kits as such are inexpensive and easy to perform which would be very beneficial for rural areas and developing countries, where expertise, infrastructure and resources are limited.
